# Plant genetic transformation: achievements, current status and future prospects

**DOI:** 10.1111/pbi.70028

**Published:** 2025-03-07

**Authors:** Peilin Wang, Huan Si, Chenhui Li, Zhongping Xu, Huiming Guo, Shuangxia Jin, Hongmei Cheng

**Affiliations:** ^1^ Academician Workstation, National Nanfan Research Institute Chinese Academy of Agricultural Sciences Sanya China; ^2^ Biotechnology Research Institute/Key Laboratory of Agricultural Microbiome (MARA) Chinese Academy of Agricultural Sciences Beijing China; ^3^ Tobacco Research Institute Chinese Academy of Agricultural Sciences Qingdao China; ^4^ Hubei Hongshan Laboratory, National Key Laboratory of Crop Genetic Improvement Huazhong Agricultural University Wuhan China

**Keywords:** plant regeneration, regeneration factor, hormone regulation, plant genetic transformation, single‐cell and spatial transcriptome

## Abstract

Regeneration represents a fundamental biological process wherein an organism's tissues or organs repair and replace themselves following damage or environmental stress. In plant systems, injured tree branches can regenerate adventitious buds and develop new crowns through propagation techniques like cuttings and canopy pruning, while transgenic plants emerge via tissue culture in genetic engineering processes intimately connected to plant regeneration mechanisms. The advancement of plant regeneration technology is critical for addressing complex and dynamic climate challenges, ultimately ensuring global agricultural sustainability. This review comprehensively synthesizes the latest genetic transformation technologies, including transformation systems across woody, herbaceous and algal species, organellar genetic modifications, crucial regeneration factors facilitating Agrobacterium‐mediated transformations, the intricate hormonal networks regulating plant regeneration, comparative analyses of transient transformation approaches and marker gene dynamics throughout transformation processes. Ultimately, the review offers novel perspectives on current transformation bottlenecks and proposes future research trajectories.

## Introduction

Gene technology involves transferring genes with known functional characteristics—such as high yield potential, stress resistance, disease and pest tolerance and enhanced nutritional profiles—into target organisms through advanced scientific methodologies. This process enables recipient organisms to acquire novel functional attributes while preserving their original genetic foundations (Atkins and Voytas, 2020; Azizi‐Dargahlou and Pouresmaeil, 2024). Numerous global nations perceive genetically modified biotechnology as a strategic developmental approach, positioning it as a critical mechanism for securing technological leadership and enhancing agricultural competitiveness (Hug, 2008; Kleter *et al*., 2001).

Typically, factors influencing gene receptor selection encompass the gene transformation mode, receptor regeneration capacity, physiological state, regeneration pathways and potential somatic cell variations. The primary limitation in gene transfer remains the receptor's regenerative capability, as establishing a high‐frequency plant regeneration system represents a fundamental prerequisite for successful genetic transformation (Li *et al*., 2019; Niazian, 2019). Many genetically modified crops emerge through *Agrobacterium*‐mediated genetic transformation and plant regeneration processes—including organogenesis and somatic embryogenesis—which are characteristically time‐intensive and heavily genotype‐dependent (Chen *et al*., 2022b; Hollingworth *et al*., 2003).

Regeneration fundamentally describes the process by which biological tissues or organs repair and replace themselves following damage or stress. Plants' remarkable regenerative capabilities find extensive application in agricultural and horticultural techniques, including cutting propagation, grafting and tissue culture methodologies (Ikeuchi *et al*., 2019; Perez‐Garcia and Moreno‐Risueno, 2018). Cellular totipotency and pluripotency serve as the cytological foundation of plant regeneration. The intricate mechanism of reprogramming plant somatic cell fate through hormonal interactions—enabling development into independent plants or organs via cell division and differentiation—constitutes the theoretical basis for developing efficient plant regeneration strategies (Bidabadi and Jain, 2020; Su *et al*., 2021).

Higher plant organs originate from shoot apical meristem (SAM), root apical meristem (RAM) and lateral meristems. Meristematic stem cells possess the unique capability to maintain their population through cell division while simultaneously differentiating into diverse tissue and organ cell types, thereby constructing complex biological structures (Lee *et al*., 2013; Song *et al*., 2021; Zhang *et al*., 2023). Consequently, these cellular reservoirs represent the fundamental tissue and organ production source in higher plants, embodying the cytological basis for plants' potentially infinite developmental capacity. Enhancing crop regeneration efficiency is a critical objective in plant tissue culture and cellular engineering domains (Qin, 2023; Xu *et al*., 2019).

Contemporary research has identified a specific class of genes that actively promote plant regeneration during transformation processes (Chen *et al*., 2022a; Jha *et al*., 2020; Uchida and Torii, 2019). Strategic gene regulation enables the development of materials with heightened genetic transformation efficiency, presenting significant implications for accelerating plant transformation methodologies.

Plant transgenic technology has substantially contributed to human societal development and economic progression. Since the first genetically modified crop's successful generation in 1983, researchers have developed over 200 transgenic plants spanning 35 botanical families. Following the initial field trial approvals in 1986, thousands of transgenic plants have undergone extensive field evaluations worldwide (Bonny, 2016; Kumar *et al*., 2020; Oliver, 2014). Currently, transgenic technologies encompass a diverse array of plant species, includingrice (*Oryza sativa* L.) (Christou, 1997; Nishimura, 2020), corn (*Zea mays* L.) (Ishida *et al*., 2007; Raji *et al*., 2018), wheat (*Triticum aestivum* L.) (Wang *et al*., 2020; Yu *et al*., 2024), tomatoes (*Solanum lycopersicum* L.) (Guo *et al*., 2012; Ruf and Bock, 2021), cotton (*Gossypium* L.) (Ge *et al*., 2023; Zhang, 2019), soybeans (*Glycine max* L.) (Dubald *et al*., 2014; Zhang *et al*., 2022), peanuts (*Arachis hypogaea* L.) (Huai *et al*., 2023; Raul and Sinharoy, 2022), lettuce (*Lactuca sativa*) (Ismail *et al*., 2016; Lelivelt *et al*., 2005), rapeseed (*Brassica napus* L.) (Bates *et al*., 2017; Calabuig‐Serna *et al*., 2023), cabbage (*Brassica rapa*) (Hu *et al*., 2019; Neumann *et al*., 2020), woody plants: pine (*Pinus* L.) (Grant *et al*., 2004; Liu *et al*., 2020), tea tree (*Camellia sinens* L.) (Fizikova *et al*., 2024; Furukawa *et al*., 2020), coconut trees (*Cocos nucifera* L.) (Nguyen *et al*., 2015), *Brachypodium distachyon* L. (Chen *et al*., 2019; Soulhat *et al*., 2023), violet (*Matthiola incana* L.) (Ghorbanzade and Ahmadabadi, 2015), blue‐green algae (*Cyanobacteria*) (Cassier‐Chauvat *et al*., 2021; Vioque, 2007) and *Chlamydomonas reinhardtii* (Matsuo and Ishiura, 2011; Scaife *et al*., 2015).

Multiple plant species demonstrate the capacity for regenerating complete organisms from isolated mesophyll protoplasts. This extraordinary capacity for plant cell regeneration not only holds significant promise for far‐reaching applications in agriculture and horticulture but also finds extensive utilization in gene transformation processes (Liao and Wang, 2023; Ramírez‐Mosqueda, 2022).

Single‐cell sequencing has emerged as a breakthrough biotechnology, garnering substantial interest in scientific and medical domains (Bawa *et al*., 2022; Hwang *et al*., 2018; Jovic *et al*., 2022; Shaw *et al*., 2021). This technology enables comprehensive life science research at the cellular level, facilitating high‐throughput genomic, transcriptomic and epigenomic analyses while enabling sophisticated cell population classification and mapping (Cui *et al*., 2024; Xu *et al*., 2021).

This review systematically examines plant genetic transformation from multiple perspectives, synthesizing insights into transformation technologies, regeneration‐promoting genes and the pivotal role of single‐cell sequencing in identifying regenerative mechanisms. By elucidating the molecular foundations of plant regeneration and cellular totipotency, we aim to establish theoretical frameworks for developing universal plant regeneration methodologies. Ultimately, our objective is to elevate molecular breeding technologies, enabling the expeditious cultivation of high‐yield, multi‐resistant, environmentally adaptive crops that ensure global agricultural sustainability.

## The method of transformation

**Figure 1:**
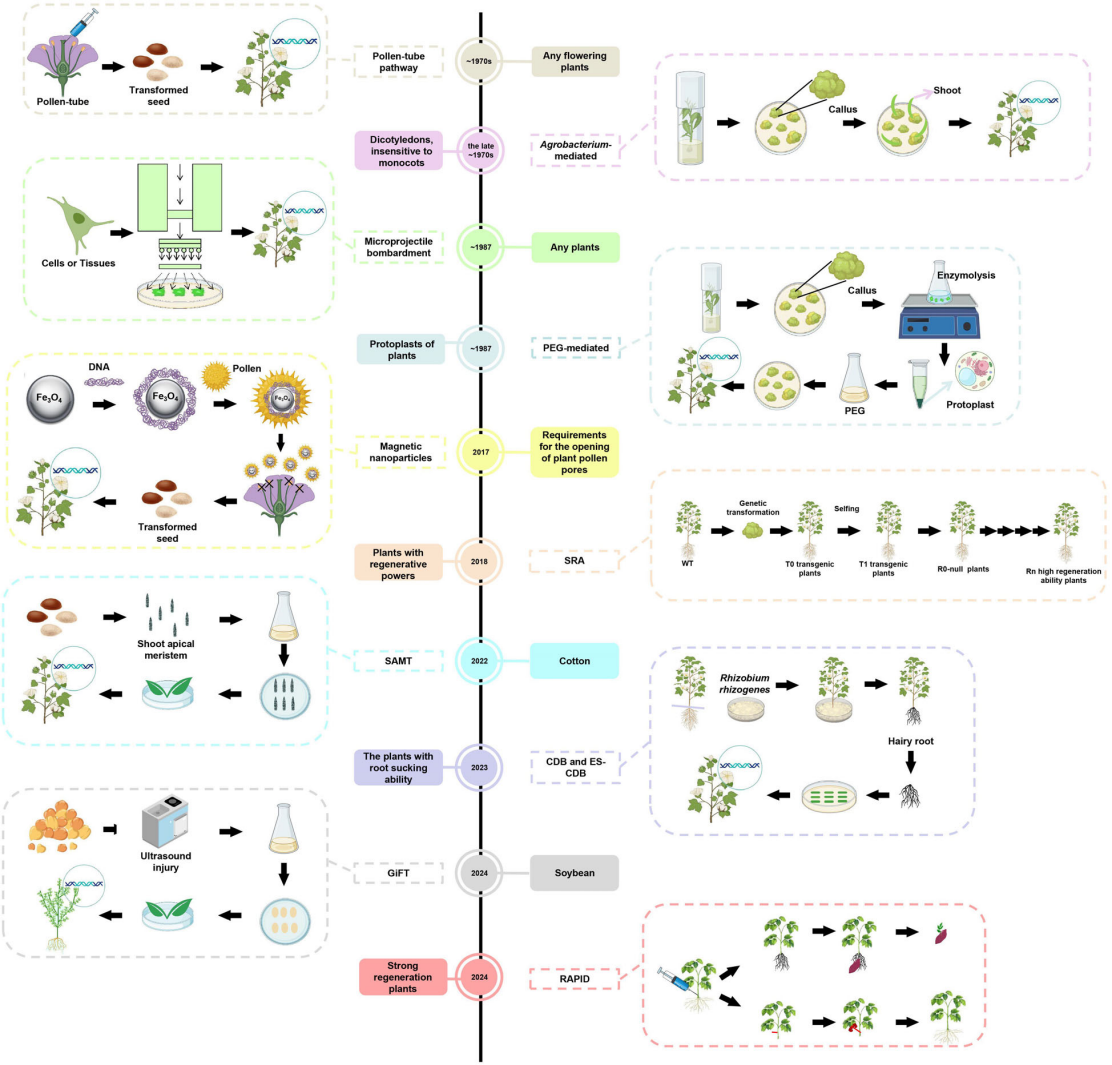
Timeline of the development of genetic transformation technology. The dotted boxes represent the key steps of the method.

Plant genetic transformation technologies can be broadly classified into two categories: direct gene transfer methods and bio‐mediated transformation methods. Direct gene transfer includes techniques such as microprojectile bombardment, protoplast methods, liposome‐mediated transfer, the pollen tube pathway, electroshock conversion and PEG‐mediated transformation, with microprojectile bombardment being the most representative. On the other hand, bio‐mediated transformation predominantly includes *Agrobacterium*‐ and virus‐mediated methods, with *Agrobacterium*‐mediated transformation being particularly popular due to its simplicity, low cost and high transformation efficiency. It has become a widely adopted technique for the genetic transformation of dicots (Anand and Jones, 2018; Su *et al*., 2023). Recently, new technologies have been developed to enhance the efficiency of crop genetic transformation, although they come with certain limitations (Figure 1).

### Pollen‐tube pathway transformation

In 1980, Hess demonstrated that pollen grains could absorb exogenous DNA (Hess, 1980), marking the inception of pollen tube channel technology. This technology is grounded in the hypothesis proposed by Chinese scholar Zhou Guangyu in the 1970s, which suggested the potential for DNA fragment hybridization during distant hybridization in plants (Jian *et al*., 2012). After pollination, the ovary is injected with a DNA solution containing the target gene. The pollen tube, formed during fertilization, acts as a conduit for the foreign DNA to enter the fertilized egg cell, where it integrates into the genome of the recipient cell, leading to the development of a transgenic individual as the zygote matures. The major advantage of this method is that it bypasses the need for tissue culture and artificial plant regeneration, making the technology simple and accessible without the need for a well‐equipped laboratory. This method has been widely adopted by researchers and has been successfully applied to important crops such as cotton (Huang *et al*., 1999; Wang *et al*., 2013; Zhao *et al*., 2021; Zhou *et al*., 1983), melon (Hao *et al*., 2011; Yang *et al*., 2009; Zhang and Luan, 2016), soybean (Shou *et al*., 2002; Yang *et al*., 2011), wheat (Martin *et al*., 1992) and corn (Zhang *et al*., 2005). In a recent study aimed at developing a genetic transformation system for *Paphiopedilum* Maudiae, it was found that the optimal period for exogenous DNA transformation was prior to the fusion of sperm and egg, during which ovary injections resulted in a high transformation efficiency of up to 2.54% (Luo *et al*., 2020).

### *Agrobacterium*‐mediated transformation

*Agrobacterium tumefaciens‐*mediated transformation is one of the most widely used methods for gene transfer in plants. This technique was first reported in the late 1970s, when Mayer used *Agrobacterium* to infect potato tissues, successfully transferring bacterial genes into the plant's cells. This pivotal research laid the foundation for developing *Agrobacterium*‐mediated transgenic technologies (Krenek *et al*., 2015). *Agrobacterium* is naturally capable of infecting most dicots and gymnosperms through chemotaxis. The cells at the injury site secrete phenolic compounds, which attract *Agrobacterium* to these areas, inducing the formation of crown galls or hair‐like roots. The bacterium's Ti plasmid (Tumour‐inducing plasmid) has the unique ability to integrate foreign DNA into plant chromosomes and synchronize gene expression with the plant's endogenous genes. The *Agrobacterium* Ti plasmid transformation system is the most extensively studied, well‐understood and widely applied method in plant genetic engineering, applicable to various species, including woody plants, herbaceous plants and algae. A recent study developed a rapid, high‐throughput, genotype‐independent soybean transformation method called GiFT (Genotype‐independent Fast Transformation) (Zhong *et al*., 2024). This approach involves using germinated seeds as explants, applying ultrasonic damage, incubating with *Agrobacterium* suspension and then screening the resulting transgenic seedlings with herbicides during planting. This method requires minimal tissue culture, yields transgenic plants quickly and produces stable transgenic lines with high inheritance potential (Zhong *et al*., 2024).

### Microprojectile bombardment

Microprojectile bombardment, also known as biolistic transformation, was first introduced by Klein *et al*. (1987). They used ultrasound to coat tungsten particles (0.2–2.0 μm) with exogenous DNA and propelled them using the dynamics of a gun to penetrate the cell wall and protoplast membrane. This allows for integrating foreign DNA into the plant genome, enabling the expression of transgenes in intact tissues. However, gene gun conversion rates are generally lower than *Agrobacterium*‐mediated transformation and the method is more costly. Furthermore, the gene gun method often produces high chimera ratios and poor genetic stability. Additionally, foreign genes integrated through this technique are usually present in multiple copies, which can lead to abnormal gene expression and potential co‐suppression (Dong and Ronald, 2021).

### PEG‐mediated transformation

PEG‐mediated transformation operates similarly to PEG‐induced protoplast fusion, but while fusion occurs between protoplasts, in transformation, it occurs between protoplasts and DNA. PEG, a polymer with a slightly negative charge, interacts with the cell membrane's phospholipid bilayer, disrupting the cell's surface potential and facilitating the uptake of plasmids or T‐DNA into the cell (Klebe *et al*., 1983). Penttila pioneered PEG‐mediated transformation in *Trichoderma reesei* protoplasts in 1987 and subsequent researchers have built upon his work. When combined with high concentrations of divalent calcium ions (Ca^2+^) and alkaline pH, PEG promotes the precipitation of DNA onto the protoplast membrane or facilitates endocytosis, allowing foreign DNA to enter the protoplast and integrate into the plant's chromosomes. PEG‐mediated transformation has become a standard method for plant genetic transformation and has also been used in fungi and bacteria (Batth *et al*., 2021; Díaz and Koop, 2021; Granado *et al*., 1997; Leng *et al*., 2024; Lu *et al*., 1994; Shuster and Bindel Connelley, 1999; Zienkiewicz *et al*., 2019).

### Magnetic nanoparticles

Magnetic Fe₃O₄ nanoparticles offer a promising approach for gene delivery to pollen under the influence of an external magnetic field. This method enables the direct acquisition of transgenic seeds through artificial pollination via the plant's natural reproductive process. The resulting transgenic offspring can be selected for stable inheritance (Zaragosa *et al*., 2024; Zheng *et al*., 2023). Combining nano‐magnetic transformation with pollen‐mediated techniques, this method addresses the limitations of traditional genetic transformation methods, particularly those involving tissue culture regeneration and host adaptability. It enhances genetic transformation efficiency, shortens the cultivation cycle of transgenic plants and supports high‐throughput and multi‐gene transformations. This approach holds significant potential for advancing crop genetics, synthetic biology and bioreactor applications. It has already been successfully applied to crops such as cotton, maize, pumpkin and pepper (Wang *et al*., 2022a, 2022b, 2022c; Zhao *et al*., 2017). Although there is some controversy about the applicability of the method and further refinement is needed to accommodate a wider range of plant transformations (Vejlupkova *et al*., 2020), it is nevertheless a revolutionary delivery method.

### Successive regeneration acclimation (SRA) strategy

Genetic modification of crops through *Agrobacterium*‐mediated transformation often involves time‐consuming and genotype‐dependent plant regeneration processes, such as organogenesis and somatic embryogenesis. For instance, cotton transformation is hindered by low regeneration efficiency, with the entire process requiring up to one and a half years (Jin *et al*., 2006a, 2006b); this leads to long transformation cycles (Jin *et al*., 2012; Li *et al*., 2019). To overcome these challenges, the SRA strategy has been developed to improve transformation efficiency. By using regenerated seeds from somatic embryogenesis as recipient material for subsequent tissue cultures, this process can be repeated to achieve higher genetic transformation rates. Researchers suggest that epigenetic modifications, particularly low methylation during SRA, may activate somatic embryonic genes, further enhancing plant regeneration (Li *et al*., 2019).

### Genetic transformation using *Agrobacterium rhizogenes*

*Agrobacterium* has long been employed for plant genetic transformation, and recent studies have highlighted the potential of *Agrobacterium rhizogenes* in transforming certain plant species (Sharma *et al*., 2021; Wang *et al*., 2024a; Yan *et al*., 2023). Some plants possess unique root regeneration abilities, and *A. rhizogenes* facilitates efficient transformation in these species. For example, *A. rhizogenes* has been used to transfer target genes into the hairy roots of apple (*Malus domestica*) and kiwifruit (*Actinidia chinensis*), achieving a high transformation efficiency of 78.8%. These hairy roots were subsequently regenerated into shoots, with a conversion rate of 3.3%. This approach can potentially be applied to other tree species, broadening the scope of genetic research and breeding programs (Liu *et al*., 2024).

A novel ‘cut‐dip‐budding’ (CDB) transformation method has been developed for species with root‐sucking abilities (Cao *et al*., 2023a). This technique involves inducing and transforming capillary roots from incision sites of plant explants with *Agrobacterium tumefaciens*. The CDB method, which involves cutting plant rhizomes under non‐sterile conditions and cultivating them on vermiculite, offers a wide application for species with root‐sucking abilities. Although initially verified in species such as *Taraxacum kok‐saghyz*, *Coronilla varia*, sweet potato, and certain woody plants (*Ailanthus altissima*, *Aralia elata* and *Clerodendrum chinense*), CDB holds promise for expansion across additional species (Cao *et al*., 2023b). Based on the CDB method, a simplified TKS program (ES‐CDB) has been developed that omits capillary root formation, saving time and labour. Though current limitations exist regarding the species and explants used, this method could revolutionize the genetic improvement of more plant species in the future (Cao *et al*., 2023b).

The RAPID method, applied to the highly regenerative plant sweet potato, involves testing various delivery methods to optimize transformation efficiency. Through stem injections of *Agrobacterium*, transgenic plants can be quickly generated. The method has been optimized for *Agrobacterium* strain selection, infection concentration, and the use of chemical agents, allowing the delivery of reporter genes and genetic editing tools. Genetic and cytological analyses have confirmed the rapid regeneration of newly transformed organs, enabling the fast acquisition of stable transgenic plants. Currently, the RAPID method is primarily applied to asexual reproduction in resource and cash crops such as sweet potato (*Ipomoea batatas*), potato (*Solanum tuberosum*) and bayhops (*Ipomoea pes‐caprae*) (Mei *et al*., 2024). By overcoming the limitations of traditional methods, the RAPID approach promises to facilitate the genetic improvement of more plant species and accelerate the development of new traits and improved germplasm for resource plants and cash crops.

### Genetic transformation of woody plants

Depending on the length of time for the entire life cycle, herbaceous plants are generally divided into annual, biennial and perennial plants. In addition to some conventional food crops, the genetic transformation of herbaceous plants is mostly mediated by *Agrobacterium* (Longo *et al*., 2006; Chen *et al*., 2019). Woody plants are invaluable both ecologically and economically, providing essential resources such as energy, building materials, food, carbon storage, biodiversity and climate regulation. However, they face significant challenges from pest invasions, disease and climate change. Coupled with the growing demand for sustainable forest products, there is an urgent need to integrate biotechnologies such as genetic modification and gene editing to improve forest resilience and sustainability (Strauss *et al*., 2019; Xu and Zhai, 2021). Despite the promise of these technologies, the genetic transformation of woody plants is complex due to their intricate genomes. Transformation efficiencies vary widely across species and even between genotypes of the same species. While *Agrobacterium*‐mediated transformation is the most commonly used method for woody plants, its efficiency is not uniform (Xu *et al*., 2020). For example, sweet cherry (*Prunus avium* L.) shows a transformation efficiency of just 1.2% in leaves (Zong *et al*., 2018), while poplar (*Populus* L.) achieves 32.18% in young leaves (Song *et al*., 2019). Nevertheless, Agrobacterium transformation does not work universally. For instance, the tea tree (*Camellia sinensis*) is genetically incompatible with common *Agrobacterium* strains, and the regeneration system limitations hinder its use in tea plant genetic research (Furukawa *et al*., 2020).

Conifers, which are valued for ornamental, edible and medicinal purposes, present additional challenges due to their biological characteristics that complicate somatic embryogenesis (SE) and *de novo* organogenesis (DNO) from *in vitro* cultured tissues (Klimaszewska *et al*., 2005; Tang and Newton, 2003). To overcome these limitations, micropropagation, alongside cutting‐based rooting techniques, has emerged as the most effective method for large‐scale propagation of elite conifer varieties. Micropropagation involves the culture of cells, tissues or organs in artificial media enriched with plant growth regulators (PGRs) under sterile, controlled conditions (Brunoni *et al*., 2019; Cardoso *et al*., 2018).

Considerable progress has been made in *Agrobacterium*‐mediated genetic transformation for conifer species such as *Pinus*, *Picea* and *Larix*, with a focus on embryogenic cultures and regeneration through somatic embryogenesis (Bonga *et al*., 2010; Klimaszewska *et al*., 2009; Trontin *et al*., 2007; Zhou *et al*., 2022). Successful initiation of embryogenic cultures from adult trees has been reported in a few cases, including from *Picea abies* (Harvengt *et al*., 2001), *Picea glauca* (Klimaszewska *et al*., 2011), *Pinus kesiya* (Ravindra *et al*., 2004), *Pinus patula* (Malabadi and Van Staden, 2005), *Pinus roxburghii* (Malabadi and Nataraja, 2006), *Pinus wallichiana* (Malabadi and Nataraja, 2007a) and from secondary needles in *Pinus roxburghii* (Bueno *et al*., 2021; Malabadi and Nataraja, 2007b). Despite these successes, rooting of tissue culture seedlings remains a major obstacle. For instance, species like *Populus tomentosa* (Nivas and Souza, 2014) and certain *Catalpa* and *Eucalyptus* species face challenges in adventitious root formation (Abiri *et al*., 2020). Root development is a complex, multifactorial process influenced by age, environmental factors, genetic traits, mineral nutrition and plant hormones (Abiri *et al*., 2020; Wang *et al*., 2014).

While several woody plants have established transformation systems, these processes remain time‐consuming, especially for species with long growth cycles. A promising alternative involves using *Rhizobium rhizogenes*‐mediated hairy root systems, offering a more efficient path to stable inheritance. For example, a transformation system for pigeon pea, an important woody food crop, has been optimized by adjusting variables such as Agrobacterium strain selection, bacterial concentration, injection site and seedling age, resulting in high transformation rates. This method has also been successfully extended to other economically important species, including herbs (*H. manihot* L.), shrubs (*C. cajan*) and trees (*M. domestica*) (Meng *et al*., 2019). The CDB delivery system, previously mentioned, has also demonstrated effectiveness for two herbaceous plants (*Taraxacum kok‐saghyz* and *Coronilla varia*), a tuberous root plant (sweet potato) and three woody species (*Ailanthus altissima*, *Aralia elata* and *Clerodendrum chinense*) (Cao *et al*., 2023a). By leveraging *Agrobacterium rhizogenes*‐mediated transformation, this system allows for the efficient and stable transformation of root cell fate and has been successfully applied to fruit trees such as cherry, blueberry and jujube (Liu *et al*., 2024). These advancements present new opportunities for the genetic improvement of woody plants, especially those with complex transformation processes.

### Genetic transformation of algae

Algae, among the oldest and simplest organisms, exhibit remarkable adaptability and diversity. As primary producers, they play a crucial role in ecosystems (Pessoa *et al*., 2023). Through metabolic processes, algae synthesize numerous bioactive compounds such as proteins, fatty acids, vitamins, pigments, sterols, polyphenols, flavonoids, terpenoids and polysaccharides (Khoo *et al*., 2019; Li‐Beisson *et al*., 2015; Pereira and Valado, 2023; Rosales‐Mendoza *et al*., 2020). These versatile organisms are utilized across various industries, including medicine, food production, environmental conservation, industrial manufacturing, soil improvement and ecosystem restoration.

Microalgae, in particular, have garnered significant attention as hosts for genetic engineering. Compared to terrestrial plants, their simpler structure, faster reproduction, higher photosynthetic efficiency and greater yield per unit area make them ideal for genetic manipulation (Chia *et al*., 2022). Despite these advantages, efficient genetic transformation remains a challenge for most algal species. Current methods include the glass‐bead technique, electroporation, gene gun bombardment and *Agrobacterium*‐mediated transformation. For cell wall‐deficient algae, the glass‐bead method is preferred due to its efficiency, low cost and simplicity (González‐Fernández *et al*., 2023; Ortiz‐Matamoros *et al*., 2018).

*Chlamydomonas reinhardtii* is often referred to as ‘photosynthetic yeast’ due to its ease of cultivation, rapid growth cycle and high photosynthetic efficiency. As a model species in cell and molecular biology (Wang *et al*., 2024d), it has been instrumental in advancing genetic transformation techniques. In 1988, the first successful transformation of *C. reinhardtii* involved introducing a wild‐type *atpB* gene into an *atpB*‐defective chloroplast line via gene gun bombardment, restoring the recipient cells' photosynthetic ability (Boynton *et al*., 1988). Subsequent research has focused on using *C. reinhardtii* to express recombinant proteins, leveraging its unique features: (i) low production costs and high efficiency, (ii) *Chlamydomonas*, as a eukaryotic organism, can carry out accurate post‐translational processing modification of proteins and (iii) a chloroplast genome resembling prokaryotic genomes, allowing it to express prokaryotic proteins effectively (Wang *et al*., 2019a; Zhang *et al*., 2021).

Cyanobacteria, as photosynthetic prokaryotes, offer distinct advantages for genetic transformation. They exhibit rapid photosynthetic growth, possess a smaller genome, lack subcellular compartments and are not subject to epigenetic gene silencing, making them easier to manipulate genetically. Additionally, cyanobacteria have a wealth of genetic and computational tools compared to microalgae.

The transformation of cyanobacteria began in 1970 when exogenous DNA uptake was first observed in *Synechococcus elongatus* (Shestakov and Khyen, 1970). In 1980, it was demonstrated that *Agmenellum quadruplicatum* strain PR‐6 could be transformed with exogenous genes (Stevens and Porter, 1980). Over subsequent decades, numerous genes were successfully introduced into cyanobacteria (Kukil *et al*., 2023; Yu *et al*., 2013; Zang *et al*., 2007). Notably, some cyanobacteria possess a natural transformation system, enabling direct uptake of exogenous DNA during the logarithmic growth phase without additional treatment (Nies *et al*., 2023; Pope *et al*., 2020). However, this ability is predominantly observed in species from the genera *Synechococcus* and *Synechocystis* (Wei *et al*., 2004).

CRISPR/Cas9 has emerged as a transformative tool for cyanobacterial metabolic engineering. For example, the deletion of the *glgC* gene, which encodes glycogen synthesis, demonstrated the technology's potential for improving cyanobacterial bioproduction of succinate (Li *et al*., 2015, 2016). CRISPR‐based approaches have since been successfully applied to several cyanobacterial species, including *Synechocystis* sp. PCC6803, *Synechococcus elongatus* PCC7942, *S. elongatus* UTEX2973 and *Anabaena* sp. PCC7120 (Behler *et al*., 2018; Lu *et al*., 2022; Sengupta *et al*., 2022).

Given its significant potential for metabolic engineering, CRISPR/Cas9 is expected to remain a focal point for future research. Investigations into the mechanisms of various natural CRISPR systems across diverse microorganisms will likely expand its applications and efficacy in genetic engineering.

### Organelle genome transformation

Nuclear genome transformation has become a widely utilized technique across most economically significant plant species (Wang *et al*., 2024b). However, it presents several limitations, such as unpredictable expression of target genes, alterations in genome structure caused by random insertions and gene silencing due to the arbitrary integration sites of transfer DNA (Meyers *et al*., 2010). Plant cells house three types of DNA‐containing organelles: the nucleus, plastids and mitochondria. Among these, chloroplast genetic transformation operates independently of nuclear transformation, offering an innovative approach to introducing exogenous genes into plants. Chloroplast transformation boasts numerous advantages, including high efficiency in exogenous gene expression, site‐specific integration, elimination of positional effects, genetic stability and prevention of gene drift through pollen (Fuentes *et al*., 2018; Maliga, 2004). This plastid transformation technique has been successfully extended to more than 20 species of flowering plants (Ahmad *et al*., 2016; Yu *et al*., 2020).

Researchers have employed chloroplast transformation to introduce a CO₂‐concentrating mechanism from cyanobacteria into transplastomic plants to enhance carbon fixation and photosynthesis. Additionally, transferring a foreign *Rubisco* from *Synechococcus elongatus* PCC7942 into tobacco chloroplasts enabled the functional assembly of Rubisco with improved CO₂ fixation efficiency, though it resulted in reduced growth under elevated CO₂ levels (Lin *et al*., 2014; Occhialini *et al*., 2016). Similarly, co‐expression of *Arabidopsis rbcL* and its cognate assembly chaperone RAF1 in the tobacco plastid genome yielded a twofold increase in photosynthesis compared to transplastomic lines expressing *rbcL* alone (Whitney and Andrews, 2001).

In 1988, the first successful chloroplast genetic transformation was achieved in the unicellular eukaryote *Chlamydomonas reinhardtii* (Boynton *et al*., 1988). This milestone was soon followed by the successful transformation of tobacco (*Nicotiana tabacum*), establishing that plastids could be used for genetic engineering in higher plants (Svab *et al*., 1990). Chloroplast transformation, when compared to conventional nuclear genome transformation, exhibits superior advantages, including targeted integration, genetic stability and the absence of gene drift through pollen (Fuentes *et al*., 2018; Maliga, 2004). Interestingly, despite the availability of various DNA delivery methods for chloroplasts, the gene gun remains the only reliably reproducible approach (Daniell *et al*., 2021; Jin *et al*., 2011, 2015; Jin and Daniell, 2015).

Recent advances in single‐base editing technologies for chloroplast genomes (Kang *et al*., 2021; Li *et al*., 2021b; Mok *et al*., 2022; Nakazato *et al*., 2023; Wang *et al*., 2024c; Zhang *et al*., 2024a) have circumvented some limitations of chloroplast transformation. These techniques hold great potential for crop improvement and breeding. A recent study achieved herbicide‐tolerant *Arabidopsis thaliana* V219I and A251V double mutants by editing chloroplast genes, a process less susceptible to pollen drift and contamination of wild‐type species compared to nuclear transformation (Nakazato *et al*., 2024).

The development of mitochondrial genome engineering presents another frontier in plant transformation. Despite its potential to significantly enhance mitochondrial gene functional studies, current mitochondrial transformation techniques remain challenging. These challenges stem from the lack of mitochondria‐specific marker genes and uncertainties surrounding homologous recombination efficiency (Li *et al*., 2011; Wang *et al*., 2024c). Notably, mitochondrial gene editing using TALEN targeted the *nad9* gene in tobacco mitochondria, isolating homologous mutants with single amino acid substitutions (Forner *et al*., 2022). The advent of CRISPR/Cas9 technology and stable mitochondrial transformation systems would mark a significant milestone, resolving mitochondrial disease challenges and furthering mitochondrial research.

### Transient transfection

Transient transfection is a method used to introduce DNA into eukaryotic cells. In this process, recombinant DNA is delivered into a highly infectious cell line, resulting in transient but high levels of gene expression (Trinidad *et al*., 2021). Unlike stable transfection, the transfected DNA does not need to integrate into the host chromosome and cells can be harvested relatively quickly. Although the expression is temporary and diminishes as the cells divide, this method is advantageous due to its high expression levels. It is particularly suitable for short‐term gene expression analysis, gene knockout studies, RNA silencing experiments and small‐scale protein production.

Transient transfection involves introducing foreign genes into cells using a vector. Although genetic modification is temporary, the method is widely used in molecular experiments due to its simplicity, quick turnaround time and low cost. Subcellular localization studies, whether through tobacco leaf injections or protoplast‐based methods, are common applications for studying gene expression (Wang *et al*., 2022a, 2022b, 2022c). Virus‐induced gene silencing (VIGS) is another related technique in which viruses carrying target gene fragments infect plants, leading to the degradation or methylation of mRNA from homologous genes. This results in the silencing of plant endogenous genes, which then causes changes in phenotypic or physiological indicators, enabling the study of gene functions through observed phenotypic variation (Kumagai *et al*., 1995; Zulfiqar *et al*., 2023). Compared to traditional gene function analysis methods, VIGS offers advantages such as faster results, no need to develop stable transformants and the ability to silence single or multiple genes within a gene family (Burch‐Smith *et al*., 2004).

A key component of transient expression technology is the construction of expression vectors based on viral genomes. Tobacco mosaic virus (TMV) and Potato virus X (PVX) vectors are commonly used to express large amounts of exogenous genes. However, as RNA viruses, they are less efficient at expressing multiple proteins simultaneously. The twin virus vector system improves the expression of large exogenous proteins, while the Tobacco Crisp Virus vector system is ideal for gene silencing and expression in plant roots (Creager, 2022; Wu *et al*., 2019). Recent advancements have made VIGS a high‐throughput tool capable of inducing heritable epigenetic modifications in plants via the viral genome, enabling the transient knockdown of targeted gene expression (Zulfiqar *et al*., 2023).

Transient transfection in tobacco is also used for transcriptional element cloning analysis, such as through dual luciferase reporter assays. This system uses firefly luciferase and renilla luciferase, and the fluorescence ratio generated by their expression can be used to monitor intermolecular interactions at the microscopic level (Zhang *et al*., 2024b). Bimolecular fluorescence complementation (BiFC) is another important technique for detecting protein–protein interactions (Ohad *et al*., 2007; Wang *et al*., 2023a). Additionally, novel methods based on transient transfection in tobacco leaves enable the visual validation of transcription factor–DNA interactions (Zhou *et al*., 2025).

For large‐scale production of exogenous proteins, plants serve as an economical and safe bioreactor when combined with transient expression methods. This approach is characterized by rapid production, high yields and a broad host range, making it ideal for producing important proteins such as antibodies, vaccines, growth factors, hormones and recombinant enzymes. This method is increasingly used for the industrial‐scale production of proteins in medical and scientific research (Chan and Daniell, 2015; Ruiz *et al*., 2015; Takaiwa *et al*., 2015).

Recent advancements in transient transfection using *Agrobacterium tumefaciens* have become a prominent area of research. Researchers have successfully overexpressed genes in hairy roots via *Agrobacterium tumefaciens* to study plant stress tolerance (Che *et al*., 2019; Wei *et al*., 2016), root biology and root‐pathogen interactions (Lozovaya *et al*., 2004; Mugnier and Mosse, 1987; Nuutila *et al*., 1995). This method has also enabled transient protein expression in the root system, leading to the production of significant amounts of metabolites (Kai *et al*., 2011; Verma *et al*., 2009). Additionally, scientists have employed this technique to rapidly transfect CRISPR/Cas9‐mediated editing vectors into hairy roots, facilitating the swift assessment of target site selection and editing efficiency (Nguyen *et al*., 2022; Zhou *et al*., 2022).

The primary advantage of transient transfection, compared to stable inheritance, is its remarkably short cycle time. Building on the success of transient transfection, researchers have extended this approach to include *Agrobacterium rhizogenes*‐mediated stable genetic transformation (Cao *et al*., 2023a, 2023b). This represents a promising direction for future genetic transformation research, where transient transfection may be strategically enhanced to achieve stable inheritance. This advancement will enable the efficient and rapid production of stable genetic materials, greatly accelerating progress in plant genetic engineering.

## Related genes that promote plant transformation

Transgenic technology enables the transfer of genes with known beneficial traits—such as high yield, stress resistance, disease and pest resistance, or enhanced nutritional quality—into target organisms, equipping them with new functional attributes alongside their existing genetic characteristics. This innovation has been instrumental in developing new crop varieties and products (Li, 2020; Shewry *et al*., 2008). Globally, genetically modified biotechnology is regarded as a strategic cornerstone for advancing scientific progress and strengthening agricultural competitiveness (Kleter *et al*., 2001).

Several factors influence gene receptor selection, including transformation methods, regeneration potential, receptor material's physiological state and regeneration pathway and somatic clonal variation. Among these, the receptor's regeneration ability is paramount, as the establishment of a high‐frequency plant regeneration system is crucial for successful genetic transformation (Li *et al*., 2019). Many genetically modified crops are developed through *Agrobacterium*‐mediated genetic transformation and subsequent plant regeneration (e.g., organogenesis and somatic embryogenesis). However, this process is highly time‐consuming and genotype dependent (Chen *et al*., 2022b).

**Figure 2:**
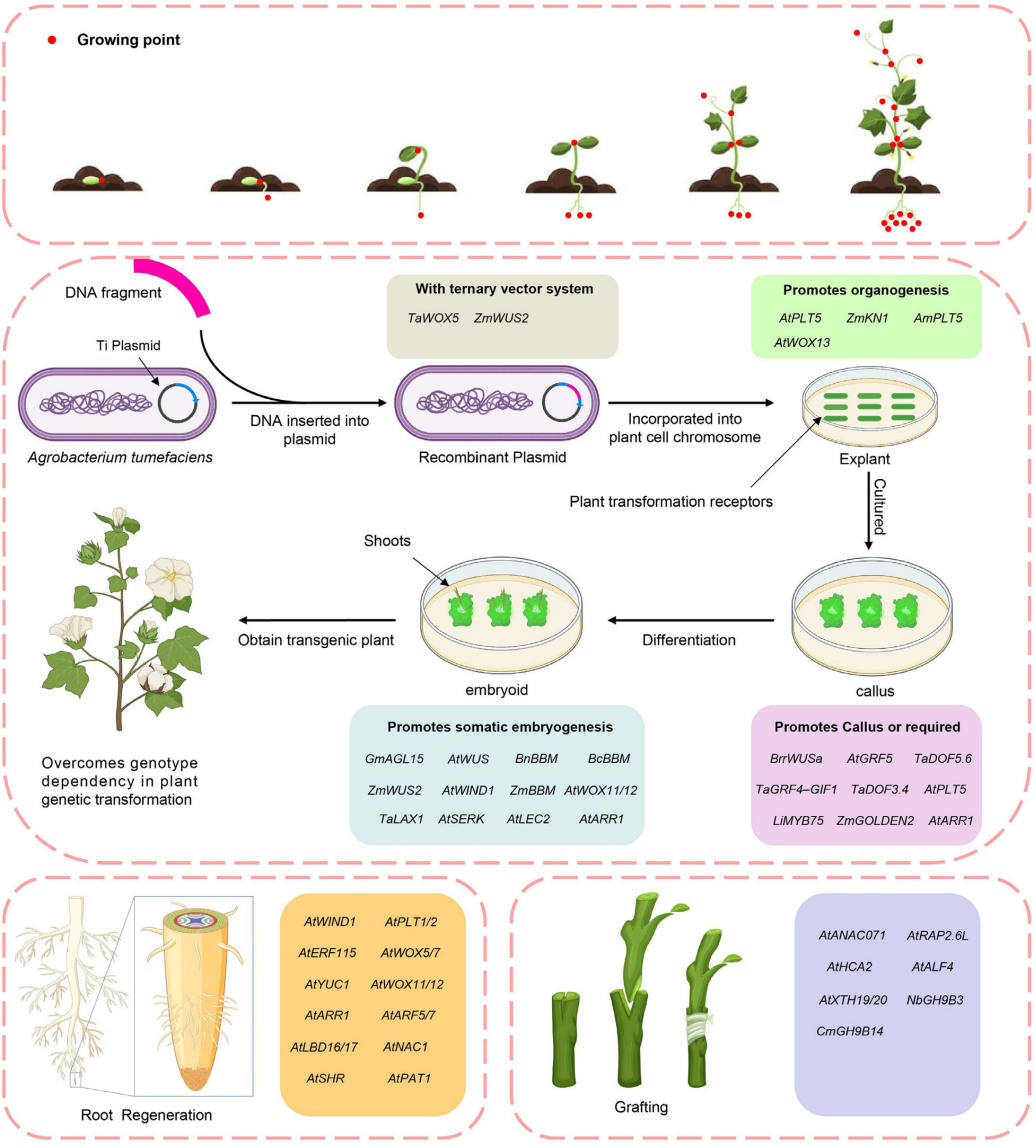
The main processes of genetic transformation and the transformation‐related genes in each step.

Recent studies have identified a class of plant genes that can enhance regeneration and improve transformation efficiency. By regulating these genes, researchers can obtain plant materials with a higher transformation efficiency, thus overcoming genotype dependency and accelerating the transformation and regeneration cycle. This breakthrough holds immense potential for improving plant species with low regeneration efficiency or those considered difficult to regenerate. Here, we summarize key genes influencing transformation efficiency during Agrobacterium‐mediated transformation and highlight their primary functional periods (Figure 2; Table 1), providing transformative solutions for enhancing genetic modification across diverse plant species.

**Table 1:** Genes associated with genetic transformation

Gene name	Gene full name	Species	Function	Notice	References
*Wox2a*	Wuschel‐like homeobox 2a	*Zea mays* L.	Induction of somatic embryogenesis and embryonic callus proliferation	No negative effects were observed in overexpressing Wox2a plants	McFarland *et al*. (2023)
*WIND1*	Wound Induced Dedifferentiation1	*Arabidopsis thaliana*	Promote de novo bud regeneration of root explants and enhance shoot regeneration		Iwase *et al*. (2015)
*BBM‐WUS2*	Baby Boom‐Wuschel2	*Zea mays* L.	Stimulation of the growth of embryogenesis tissues	The ectopic expression of *Bbm/Wus2* will affect the growth of regenerated plants and cause sterility	Lowe *et al*. (2016)
*GRF4‐GIF1*	Growth‐Regulating Factor/GIFGRF‐Interacting Factor	*Triticum aestivum*	Improve the regeneration efficiency	GRF4‐GIF1 can also function in dicot	Debernardi *et al*. (2020)
*AtGRF5*	Growth‐Regulating Factor5	*Arabidopsis thaliana*	Accelerate bud formation and significantly improve transformation efficiency	Overexpression of *GRF5*, *AtGRF6* and *AtGRF9* had a positive effect on the proliferation of transgenic callus cells	Kong *et al*. (2020)
*TaWOX5*	WUSCHEL family	*Triticum aestivum*	Improve the genetic transformation efficiency of monocots	The expression of *TaWOX5* gene did not affect callus differentiation and root development of regenerated plants	Wang *et al*. (2022)
*TaDOF5.6 TaDOF3.4*	DNA binding with one finger	*Triticum aestivum*	Improve the callus induction rate and genetic transformation efficiency		Liu *et al*. (2023)
*PLT5*	PLETHORA	*Antirhinum majus*	Promote the formation of callus and bud regeneration at the aboveground stem wound	Improve the conversion rate and germination regeneration rate and promoted the formation of embryogenic callus	Lian *et al*. (2022)
*BBM*	Baby Boom	*Malus domestica Borkh*.	Improve the transformation and regeneration efficiency of apples	The ectopic expression of *MdBBM1* enhanced cell division	Chen *et al*. (2022c)
*GRF4‐GIF1‐BBM*	Growth‐Regulating Factor/GIFGRF‐Interacting Factor‐Baby Boom	*Zea mays* L. *Triticum aestivum*	The genetic transformation efficiency of maize can be greatly improved	Improves the transformation efficiency of maize without affecting the phenotype of maize	Vandeputte *et al*. (2024)
*LiMYB75*	V‐myb avian myeloblastosis viral oncogene homologue	*Lagerstroemia indica*	Increase in the frequency of callus and the number of regenerated buds		Jiang *et al*. (2023)
*Wox13*	Wound Induced Dedifferentiation 13	*Arabidopsis thaliana*	Contribute to callus formation, organ reconnection and induced after a wound	WOX13 directly upregulates WIND2 and WIND3 to further promote cell reprogramming and organ regeneration	Ikeuchi *et al*. (2022)
*G2*	GOLDEN2	*Zea mays* L.	The callus would turn green in advance and more green spots, the differentiation efficiency and the transformation efficiency would increase	*GOLDEN2 (G2*) is a member of the GARP transcription factor superfamily, which regulates the development of chloroplasts	Luo *et al*. (2023)
*SERK*	Somstic Embrtogenesis Receptor‐like Kinase	*Arabidopsis thaliana*	Results in a 3–4‐fold increase in the efficiency of somatic embryogenesis	The encoded somatic embryogenesis receptor kinase promotes the expression of early genes in somatic embryogenesis	Hecht *et al*. (2001)
*LEC2*	Leafy Cotyledon2	*Arabidopsis thaliana*	Induces the expression of embryonic‐specific genes in vegetative cells and promotes the formation of somatic embryo‐like structures		Lotan *et al*. (1998)
*AGL15*	Agamous‐like15	*Arabidopsis thaliana*	This factor has been shown to be required for somatic embryogenesis of *M. truncatula*	*At5g61590* is essential for AGL15 to promote somatic embryogenesis. *At5g61590* effects of ethylene on somatic embryogenesis	Thakare *et al*. (2008)
*KN1*	knotted1	*Zea mays* L.	Expression of the KN1 gene increases transformation efficiency by 3‐ to 15‐fold		Hu *et al*. (2016)
*LAX1*	TaLAX Panicle1	*Triticum aestivum*	The study found that overexpression of TaLAX1 gene could significantly improve the ability of wheat sprout regeneration	Improve the regeneration ability by activating the synthesis of TaGRF4, TaGIF1, cytokinin, auxin transport‐related genes	Yu *et al*. (2024)

### Vector system‐related genes

The vector used for plant genetic transformation must be a nucleic acid molecule capable of entering host cells for replication and expression. The most commonly employed plasmids in these systems are Ti plasmids, found in *Agrobacterium tumefaciens*, and Ri plasmids, present in *Agrobacterium rhizogenes* (Moriguchi *et al*., 2001). *Agrobacterium*‐mediated transformation can be divided into four key stages: vector construction, explant infection, callus formation and differentiation into somatic embryos. Researchers have increasingly optimized delivery vectors and *Agrobacterium* strains to enhance gene delivery efficiency. For instance, the morphogenetic genes *Baby Boom* (*BBM*) and *Wuschel2* (*Wus2*) significantly improve transformation frequencies in maize while reducing genotype dependence (Lowe *et al*., 2016).

To address this issue, promoters such as PLTP (phospholipid transfer protein gene) and the auxin‐inducible Axig1 promoter were used to drive the expression of *BBM* and *Wus2*, respectively. This approach enabled genotype‐independent maize transformation without requiring callus culture. The process allowed maize embryos to directly form somatic embryos, which could then develop into fully regenerated plants with normal growth and reproduction (Lowe *et al*., 2018). Additionally, simultaneous expression of T3SS and AvrPto proteins significantly enhanced transient infection efficiency in *Arabidopsis*, promoting the development of root tumours and resistant calluses. This strategy doubled the transformation efficiency of the floral‐dip method in *Arabidopsis thaliana* (Raman *et al*., 2022).

Building on these advances, a ternary vector system combining the morphogenic regulator (MR) and CRISPR/Cas9 modules greatly improved *Agrobacterium*‐mediated delivery of genome‐editing reagents in maize, increasing transformation throughput (Zhang *et al*., 2019a). A recent strategy termed ‘altruistic transformation’ exploited the non‐cell‐autonomous function of *ZmWUS2* to generate transformed plants without retaining the *ZmWUS2* expression cassette. By using two independent *Agrobacterium* strains, one carrying the selectable marker and the other expressing *ZmWUS2*, maize embryos could be efficiently transformed (Kang *et al*., 2023).

Furthermore, a CRISPR‐based approach designed to simultaneously activate endogenous morphogenic genes and promote gene editing enhanced transformation and editing efficiencies in poplar and rice. This method yielded plants free of developmental defects, providing a valuable tool for accelerating genome engineering (Pan *et al*., 2022). Similarly, overexpression of the wheat *Wuschel‐like Homeobox5* (*WOX5*) gene significantly improved transformation efficiency in wheat, barley and maize without adverse effects on plant development, offering a promising system for monocot transformation (Wang *et al*., 2022a, 2022b, 2022c). However, in maize, *WOX5* was tested in conjunction with a ternary vector system, so its independent contribution to the reported efficiency remains to be fully understood (Wang *et al*., 2022a, 2022b, 2022c).

### Organogenesis‐related genes

Callus formation is a critical step in the plant transformation process, yet many materials cannot serve as effective receptors due to poor tissue regeneration or limited callus formation capabilities. Developmental regulators (DRs) such as *PLETHORA5* (*PLT5*), *WUSCHEL* (*WUS*) and *WUS‐P2A‐BBM* play essential roles during callus formation. In *Arabidopsis*, *AtPLT5* is primarily expressed in sink tissues, particularly in the root elongation zone, vascular tissues of leaves, floral organs like siliques and the floral abscission zone (Büttner, 2007).

In maize, expression of the transcription factor *ZmKN1*, involved in establishing and maintaining plant meristems, increased transformation efficiency by 3‐ to 15‐fold. *ZmKN1* has also been successfully used to improve shoot regeneration and transformation in tobacco and other crops (Hu *et al*., 2016). WUSCHEL‐related homeobox transcription factors, such as *AtWox13*, play pivotal roles in wound‐induced dedifferentiation and organ reconnection. In *Arabidopsis*, *AtWox13* is rapidly induced following injury and activates downstream dedifferentiation factors like *WIND2* and *WIND3*, facilitating cell reprogramming and organ regeneration (Ikeuchi *et al*., 2022).

Recently, a novel peptide termed REGENERATION FACTOR1 (REF1) was discovered to regulate wound response and regeneration. REF1 interacts with the cell surface receptor PORK1 (Pepr1/2 Ortholog Receptor‐Like Kinase1), which activates the transcription factor WIND1, a key regulator of damage response and cell reprogramming. Remarkably, the exogenous application of *REF1* improved regeneration efficiency in soybean (*Glycine max*), maize (*Zea mays*) and wheat (*Triticum aestivum*), demonstrating its significant potential for crop improvement (Yang *et al*., 2024).

Another crucial factor in tissue regeneration is the transcription factor ELONGATED HYPOCOTYL 5 (HY5), which mediates light signalling in plants. Early changes in light conditions after tissue injury influence bud regeneration efficiency, and studies have shown that HY5 mutants exhibit impaired bud regeneration. HY5 regulates the expression of key meristem formation genes, such as *WOX5*, *PLT1* and *LBD33*. Specifically, it suppresses root formation while promoting bud regeneration by activating WUS and CUC1. Early activation of photosynthesis has also been identified as critical for efficient bud regeneration (Chen *et al*., 2024).

### Formation of callus‐related genes

Explants dedifferentiate to form a callus, a crucial step in plant transformation. The viability of the callus and its ability to further differentiate into buds are pivotal stages in this process. Specific genes are essential for callus development, while others accelerate callus formation and differentiation. For instance, in turnip (*Brassica rapa* var. *rapa*), induced activation of the *BrrWUSa* gene significantly enhances regeneration efficiency, with estradiol‐inducible *BrrWUSa* transgenic plants remaining fertile and exhibiting no developmental abnormalities (Liu *et al*., 2022). In wheat, the Growth‐Regulating Factor/GIF‐Interacting Factor (*TaGRF4‐GIF1*) protein complex boosts the regeneration efficiency of transgenic plants (Debernardi *et al*., 2020). The *GRF4‐GIF1* complex has also been employed to improve transformation efficiency in multiple crops, including triticale, lettuce and rice (Bull *et al*., 2023; Qiu *et al*., 2022). In *Arabidopsis*, overexpression of *AtGRF5*, *AtGRF6* and *AtGRF9*, which belong to the same family as *GRF4‐GIF1*, promotes the proliferation of transgenic callus cells in rapeseed. Introducing *AtGRF5* or its orthologs into *Brassica napus*, soybean (*Glycine max*) and sunflower (*Helianthus annuus*) significantly enhances the genetic transformation of explant tissues. Specifically, *AtGRF5* overexpression not only stimulates transformed cell proliferation but also facilitates transgenic plant formation, accelerating bud emergence in beet (*Beta vulgaris* ssp. *vulgaris*) callus cells. Similarly, overexpression of *GRF5* in soybean and sunflower increases transformed cell proliferation and promotes transgenic bud development (Kong *et al*., 2020). Expression of AtPLT5 in snapdragon (*Antirrhinum majus*) has been shown to enhance callus formation and bud regeneration at wound sites on aerial stems, enabling stable transgene inheritance. PLT5 also improved shoot regeneration and transformation efficiencies in two Brassica rapa varieties and promoted transgenic callus and somatic embryo formation in sweet pepper (*Capsicum annum*) through *in vitro* tissue culture (Lian *et al*., 2022). GOLDEN2 (*G2*), a member of the GARP transcription factor superfamily, is another key gene involved in chloroplast development, stress responses, plant senescence and hormone signalling. Transforming rice and maize callus with *rZmG2* results in earlier greening, more green spots and improved differentiation efficiency (11.6%–48.7%), alongside enhanced transformation efficiency (8.3%–23%). RNA‐seq and RT‐qPCR analyses reveal that *rZmG2* promotes rice differentiation by upregulating chloroplast‐related genes (Luo *et al*., 2023).

### Formation of shoot‐related genes

The redifferentiation of callus into shoots involves numerous genes that promote somatic embryogenesis. Among the AP2/ERF family of transcription factors, BABY BOOM (*BBM*) is preferentially expressed in developing embryos and seeds. AP2/ERF proteins are defined by their AP2/ERF DNA‐binding domains, mediating DNA and protein–protein interactions (Boutilier *et al*., 2002). *BBM* has been shown to enhance shoot regeneration and transformation efficiency in various species. For example, ectopic expression of the *Brassica napus BABY BOOM* transcription factor overcomes genotype‐dependent bottlenecks in sweet pepper (*Capsicum annuum*), enabling efficient regeneration of transgenic plants. Transient activation of *BBM* in progeny plants induces prolific cell regeneration and produces numerous somatic embryos that readily convert into seedlings (Heidmann *et al*., 2011). Ectopic *BBM* expression in *Arabidopsis* and *Brassica* results in spontaneous somatic embryo formation and cotyledon‐like structures on seedlings, accompanied by neoplastic growth, hormone‐free explant regeneration and altered leaf and flower morphology (Boutilier *et al*., 2002). In tobacco, heterologous *BBM* expression induces spontaneous shoot and callus formation, though a cytokinin pulse is required for somatic embryogenesis (Srinivasan *et al*., 2007). Similarly, *BBM* overexpression induces somatic embryo formation from Chinese white poplar (*Populus tomentosa*) callus (Deng *et al*., 2009). In *Arabidopsis*, ectopic expression of the AP2/ERF transcription factor WOUND INDUCED DEDIFFERENTIATION1 (*AtWIND1*) in *Brassica napus* enhances shoot regeneration from root explants (Iwase *et al*., 2015). Additionally, overexpression of the SOMATIC EMBRYOGENESIS RECEPTOR‐LIKE KINASE (*AtSERK1*) gene amplifies signalling cascades, promoting early somatic embryogenesis and increasing efficiency by three to four times (Hecht *et al*., 2001). *Arabidopsis LEAFY COTYLEDON1* (*AtLEC2*) induces embryo‐specific gene expression in vegetative cells, facilitating somatic embryo‐like structure formation (Lotan *et al*., 1998). In wheat, overexpression of the *TaLAX Panicle1* (*TaLAX1*) gene enhances regeneration by activating *TaGRF4*, *TaGIF1* and cytokinin and auxin transport‐related genes, thereby improving genetic transformation and gene editing efficiencies (Yu *et al*., 2024).

### Formation of root‐related genes

Adventitious root and shoot organogenesis represent critical forms of plant regeneration following injury. Root organogenesis in *Arabidopsis* leaf explants involves two key stages. First, regenerative cells differentiate into root cells via auxin‐induced activation of *Wox11* and *Wox12*, genes associated with *Wuschel*. In the second stage, naive root cells transition into root primordia, with *Wox11/12* directly initiating *Wox5* and *Wox7* expression, key regulators of root primordium formation (Hu and Xu, 2016). Evidence indicates that callus formation requires hormone‐mediated activation of lateral and meristematic root development programmes in pericycle‐like cells (Sugimoto *et al*., 2010). Accordingly, lateral root development regulators such as AUXIN RESPONSE FACTOR7 (*ARF7*), *ARF19* and LATERAL ORGAN BOUNDARIES DOMAIN (*LBD16*, *LBD17*, *LBD18* and *LBD29*) are critical for hormone‐induced callus formation (Fan *et al*., 2012; Ikeuchi *et al*., 2013). The heterodimeric transcription factor complex ETHYLENE RESPONSE FACTOR115 (*ERF115*)‐PHYTOCHROME A SIGNAL TRANSDUCTION1 (*PAT1*) sustains meristematic function by promoting cell renewal after stem cell loss. Ectopic *ERF115*‐*PAT1* expression triggers neoplastic growth, correlating with activation of the target gene WOUND INDUCED DEDIFFERENTIATION1 (*WIND1*) (Heyman *et al*., 2016; Iwase *et al*., 2011).

## Grafting and regeneration

Grafting is a horticultural technique that joins the tissues of two plants, forming a chimeric organism through the establishment of apoplast and symplast connections. This method is extensively employed in the propagation of fruit trees and vegetables, enhancing their disease and stress resistance while preserving desirable traits. The grafting process relies primarily on tissue reunion, which involves a series of complex biological events such as wound response, cell division, cell adhesion, differentiation and vascular formation (Kurotani and Notaguchi, 2021). Cambium cells play a crucial role in this process, dividing outward to produce phloem and inward to form xylem, enabling the vascular tissues of the two plants to integrate. This ensures the upward transport of water and inorganic nutrients from the roots and the downward flow of organic compounds from photosynthesis to the roots (Feng *et al*., 2024; Thomas and Frank, 2019).

Successful grafting typically requires a close phylogenetic relationship between the scion and rootstock. Plant hormones, such as auxin, abscisic acid and cytokinin, also influence grafting outcomes (Nanda and Melnyk, 2018). For instance, soaking plant materials in 200–300 ppm naphthalene acetic acid (NAA) for 6–8 h before grafting enhances cambium activity, promoting wound healing and increasing graft survival rates. Specific genes have also been implicated in improving graft success. For example, *RAP2.6L*, regulated by ethylene, and *ANAC071*, regulated by jasmonic acid, have been shown to stimulate the formation of new tissues that restore vascular connections in grafted or injured stems (Asahina *et al*., 2011; Sharma and Zheng, 2019).

The *CmGH9B* gene family, which responds to various stimuli such as low temperature, exogenous hormones, drought and physical damage, exhibits differential expression during graft healing. In watermelon grafted onto pumpkin rootstock, both NAA and far‐red light treatment significantly upregulated *CmGH9B14* expression, facilitating scion healing. This finding provides a potential strategy for enhancing grafting success by targeting *CmGH9B14* (Zhu *et al*., 2023b).

Additionally, *NbGH9B3*, encoding β‐1,4‐glucanase, has been shown to promote interfamily grafting in tobacco (Luo *et al*., 2022). Notably, studies using *Nicotiana benthamiana* as an intermediary allowed successful grafting of tomato scions onto *Arabidopsis thaliana* or chrysanthemum rootstocks, expanding the range of viable graft combinations for creating plant chimeras (Notaguchi *et al*., 2020).

## Hormones and plant regeneration

**Figure 3:**
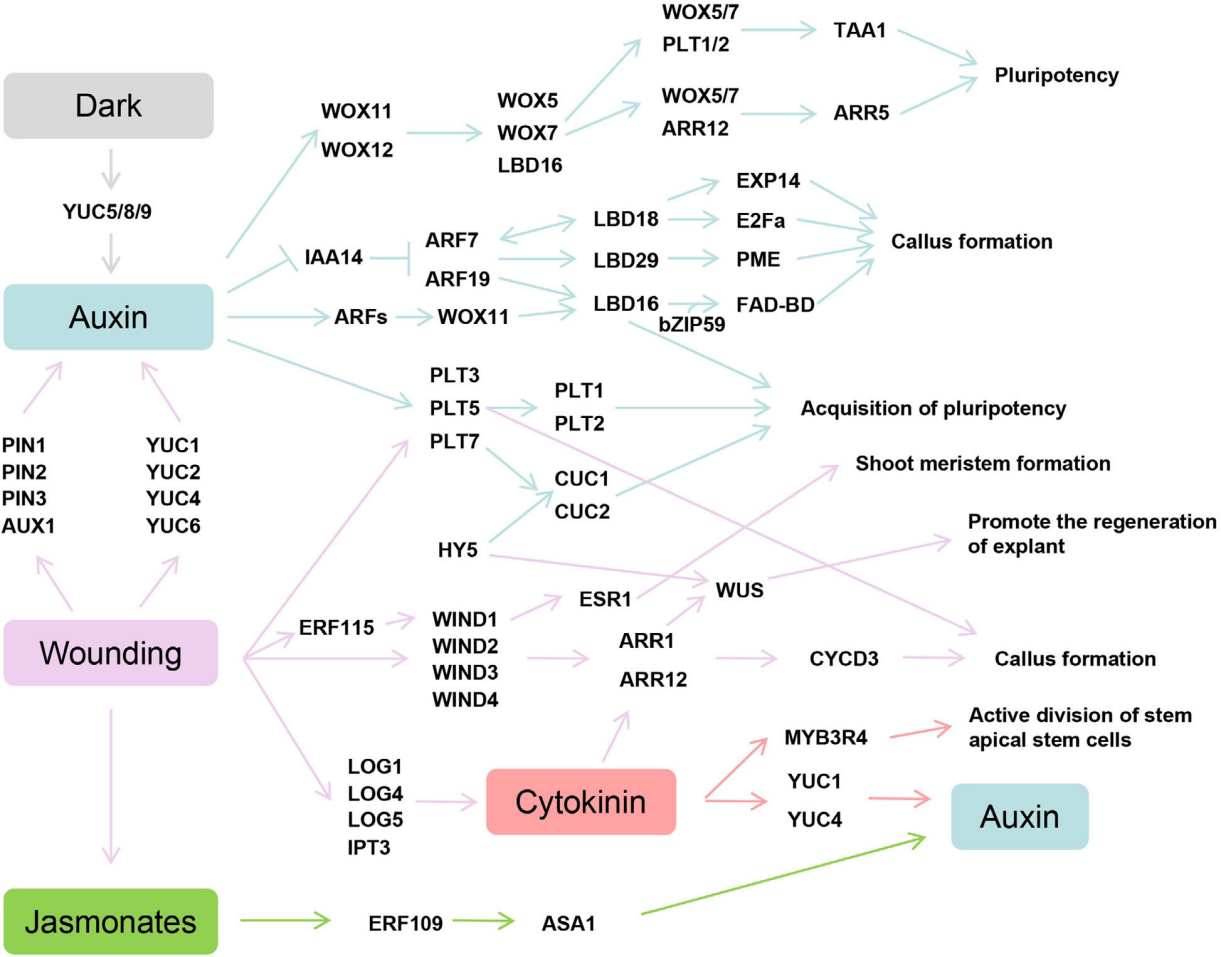
Wounding, hormone induction and regeneration‐related pathways.

Plant regeneration hinges on the fate decisions of cells, which are influenced by signals from environmental stresses or damage. Plant hormones, such as auxin and cytokinin, play critical roles in this process, guiding the determination of cell fate during regeneration (Roth *et al*., 2024). When a plant is wounded, there is often a rapid increase in various hormones, which interact and trigger the regeneration of tissues. These hormones communicate with one another, activating intricate signalling pathways that facilitate regeneration (Figure 3).

*De novo* organ regeneration refers to the ability of a plant to regenerate a complete individual from its body. Tissue culture technologies, which induce root regeneration through hormonal signals, are a prime example of *de novo* regeneration. Research has divided the *de novo* regeneration process into three general stages: the activation of primary cells, the acquisition of regenerative potential and the re‐establishment of meristems.

The activation of primary cells marks the initiation of plant regeneration. *In vitro* leaf explants detect early wound signals and environmental factors influencing their regenerative ability. Environmental signals play a pivotal role in this process. For instance, dark treatments can stimulate the expression of explant YUC5/8/9, impacting auxin levels and enhancing regenerative capacity (Matosevich *et al*., 2020). In *Arabidopsis*, wound signalling activates gene expression in damaged or surrounding tissues, sustaining auxin levels and triggering regenerative processes. Once regeneration begins, auxin synthesis is activated, and the hormone is transported to the cells with regenerative potential. These cells undergo fate transitions to form root primordium cells. These cells then differentiate through division, forming apical meristems that give rise to adventitious roots (Liu *et al*., 2018; Xu, 2018).

During *de novo* bud regeneration, callus induction medium (CIM) promotes the activation of regenerative cells, while bud induction medium (SIM) enhances the expression of genes critical for bud formation. Specifically, ARRs activate the *WUS* gene, and PLTs activate the expression of *PLT1* and *PLT2*, ultimately driving the development of *de novo* bud apical meristems (Xu, 2018). Auxin, through ARFs and *LATERAL ORGAN BOUNDARIES DOMAIN* (LBD) proteins, further activates genes, like *EXP14*, *E2Fa* and *PME2*, which promote callus formation and pluripotency acquisition. These pathways involve both *WOX11* and *LBD16*, as well as PLTs and *CUP‐SHAPED COTYLEDON* genes (*CUC1*, *CUC2*) (Ikeuchi *et al*., 2019; Liu *et al*., 2018).

Cytokinin signalling, triggered by wounding, facilitates de‐differentiation and re‐entry into the cell cycle through pathways involving *WOUND‐INDUCED DEDIFFERENTIATIONs* (WINDs) and *ISOPENTENYL TRANSFERASE* (IPT3). This process promotes the activation of *CYCD3* (Ikeuchi *et al*., 2019). Similarly, *PLT* genes induced by injury are critical for callus formation. Cytokinin regulates *YUC1* and *YUC4* expression in the *WUS* expression zone, inhibiting auxin accumulation, maintaining *WUS* gene activity and facilitating *de novo* bud regeneration. Moreover, high cytokinin concentrations increase the nuclear localization of MYB3R4, which enhances cell division and enlarges shoot meristems, contributing to the continuous response to cytokinin (Yang *et al*., 2021).

During *de novo* root regeneration, auxin triggers the expression of *WOX11* and *WOX12*, enabling primary cells to acquire regenerative potential. These factors, in turn, activate the *WOX5/7* and *LBD16* genes, which prompt primary cells to form root primordium cells and establish apical meristems *de novo*. However, it has been observed that locally synthesized cytokinin inhibits auxin accumulation, thereby affecting the activation of the *WOX5/7* gene in root primordium cells. Consequently, the balance between auxin and cytokinin plays a pivotal role in determining the direction of *de novo* organ regeneration. Specifically, the concentration ratio of exogenous cytokinin and auxin dictates the type of organ regeneration, with auxin‐induced *WOX5/7* and cytokinin‐regulated *WUS* being critical factors in this process (Motte *et al*., 2014; Sang *et al*., 2018). Further research has shown that *WOX5/7* facilitates the acquisition of pluripotency by callus mesocells through at least three mechanisms: it sustains the stem cell properties of the callus mesosphere; the *WOX5/7*‐PLT protein complex activates *TAA1*, a gene involved in endogenous auxin synthesis, which in turn increases auxin accumulation; and the *WOX5/7*‐ARR12 complex inhibits the expression of *ARR5*, breaking the negative feedback loop of cytokinin signalling and resulting in a hypersensitive state to cytokinin (Zhai and Xu, 2021). Thus, the auxin‐to‐cytokinin ratio significantly influences shoot and root regeneration during *in vitro* organogenesis. A lower auxin‐cytokinin ratio favours bud regeneration, while higher auxin concentrations promote root regeneration. Furthermore, the type of plant hormone involved also impacts phytoorganogenesis. For example, in *Arabidopsis thaliana*, IAA has been identified as the most potent auxin, capable of inducing both bud and root formation on its own. On the other hand, 2,4‐D, a synthetic auxin, is a more potent inducer of somatic embryogenesis (SE) than natural auxins or other synthetic variants (Abas *et al*., 2021).

While 2,4‐D is less efficient for bud regeneration, NAA is more effective in forming adventitious roots. Prior studies suggest that 2,4‐D is less efficiently transported by efflux mechanisms compared to IAA, which could explain its accumulation in plant cells and its stronger auxin response. This ability to accumulate may be crucial in driving effective SE induction. When used with N‐1‐naphthylphthalamic acid (NPA), an auxin efflux inhibitor, either natural or synthetic auxins can reach similar levels of effectiveness as 2,4‐D. After inducing SE or pluripotent callus formation, explants are transferred to NPA‐free medium, which allows for efficient auxin export, thus promoting the development of somatic embryos or shoot apical meristems (Karami *et al*., 2024). Overall, hormonal induction remains unpredictable, as different hormones engage in distinct pathways, and their type, concentration and the physiological state of the plant significantly influence the regeneration of tissues.

Jasmonic acid (JA), an endogenous growth regulator in higher plants, has been shown to promote stomatal closure (Zhang *et al*., 2019b), inhibit Rubisco biosynthesis (Ghorbel *et al*., 2021) and affect N and P absorption as well as glucose transport (Li *et al*., 2021a; Ruan *et al*., 2019). Moreover, JAs and salicylic acid (SA) are implicated in plant defence responses, acting as signals for activating resistance genes in response to mechanical damage, herbivore feeding or pathogen infection. Their signalling pathways are crucial for plant immunity (Attia and Alamer, 2024; Gimenez‐Ibanez *et al*., 2017). Following a wound, plants can regenerate high‐quality roots through the process of *de novo* root regeneration (DNRR), which is commonly used in agricultural practices. JA has been identified as a key wound signal that promotes DNRR in leaf explants. After leaf shedding, JA rapidly accumulates, triggering a bidirectional signalling pathway that activates the expression of *ERF109*, a gene in the AP2/EREBP transcription factor family. Just 10 min after leaf shedding, *ERF109* enhances DNRR by upregulating genes involved in auxin biosynthesis (Zhang *et al*., 2019). Auxin, a crucial hormone for cell fate determination, links early signalling events to cell fate transitions during DNRR. When auxin is transported to cells with high regenerative potential, the auxin signalling pathway directly activates the expression of *WOX11*, facilitating the transition from regenerative cells to root‐forming cells (Liu *et al*., 2022).

The study suggests that the wound signal responsible for inducing *de novo* root regeneration (DNRR) in leaf explants has at least two key effects. First, it creates a physical barrier that blocks auxin flow, leading to the highest auxin concentration at the injury site. Second, it enhances auxin biosynthesis, thereby improving the efficiency of regeneration. Specifically, injury triggers the production of jasmonic acid (JA) in leaf explants, which activates the expression of *ERF109*. This, in turn, upregulates the expression of *ANTHRANILATE SYNTHASE α1* (*ASA1*), a gene involved in tryptophan biosynthesis within the auxin production pathway, within 2 h of leaf shedding (Niyogi and Fink, 1992; Sun *et al*., 2009). Elevated *ASA1* expression enhances auxin biosynthesis, promoting root formation in leaf explants. However, after 2 h, JA levels decrease, allowing *JAZ* proteins to interact with *ERF109*, inhibiting its activity and preventing hypersensitivity to wound signals (Zhang *et al*., 2019).

Thidiazuron (TDZ, 1‐phenyl‐3‐(1,2,3‐thiadiazol‐5‐yl)) exhibits cytokinin‐like activity, but unlike other adenine‐type cytokinins, such as benzylaminopurine (BA), kinetin (Kin), and zeatin, TDZ does not contain a purine ring. Extensive research has shown that TDZ is an effective regulator in plant cell, tissue and organ culture, particularly in processes like callus induction, somatic embryogenesis and shoot organogenesis and proliferation (Dewir *et al*., 2018; Ghosh *et al*., 2018). In addition to TDZ, the regenerative capacity of plants can be further enhanced by exogenous treatments. For example, adenosine monophosphate (AMP) treatment has been found to boost plant regeneration by activating the *PLETHORA* (PLT) gene, which encodes a core regulator of pluripotency acquisition. AMP treatment has been shown to promote protoplast regeneration and increase the regenerative potential of plants such as cabbage (*Brassica oleracea var. Capitata* L. cv. Okina), tomato (*Solanum lycopersicum* L. cv. MicroTom) and Japanese whitehead Thunberg (Lee *et al*., 2023).

Reactive oxygen species (ROS) are critical as signalling molecules in plant growth and development. However, abiotic stresses can cause ROS levels to exceed optimal thresholds, resulting in cellular damage. Both excessively low and high ROS levels can impair plant growth, while maintaining ROS within an optimal range supports healthy plant development. ROS interact with various plant hormones, influencing the levels and functions of hormones such as abscisic acid (ABA), indoleacetic acid (IAA), brassinosteroids (BRs), gibberellins (GAs), salicylic acid (SA), jasmonic acid (JA) and nitric oxide (NO) (Zhu *et al*., 2017). ROS levels rise rapidly after injury, and treatments with ROS inhibitors, such as salicylhydroxamic acid (SHAM) or glutathione (GSH), have been shown to reduce the rooting ability of leaf explants (Liu *et al*., 2022). These findings underscore the importance of ROS in regulating adventitious root formation in cuttings. Furthermore, both ethylene and ROS can act as wound signals to promote DNRR after injury.

In summary, the interplay between hormone and stress signalling is fundamental to determining cell fate, creating a highly complex regulatory network of cellular reorganization. Following plant damage, electrical signals generated by nociceptive stimuli can be rapidly transmitted from leaf to leaf at a rate of several centimetres per minute (Wu *et al*., 2024). During the regeneration and repair of plant wounds, various hormones coordinate with each other, triggering distinct signalling cascades that regulate the expression of regeneration‐related genes. This allows plants to rapidly repair tissues, regenerate organs and even restore the entire organism.

## Selectable marker gene during transformation

Following genetic transformation to obtain transformants, the process of screening positive transformants is labor‐intensive and time‐consuming. This typically involves planting, collecting tissue samples, preparing genomic DNA and PCR detection of eukaryotic resistance or target genes, followed by fragment sequencing. To facilitate screening and detection of transgenic plants, researchers use selective agents, with selection based on the resistance genes carried by the transgenic plants (Table 2). Commonly used screening agents include kanamycin, G418 (geneticin), hygromycin B, spectinomycin and gentamicin. Among these, kanamycin was the first widely used in plant genetic engineering. Hygromycin, a potent cell growth inhibitor, is highly toxic to many plants, so careful selection is necessary to avoid negative effects. Additionally, herbicides (such as 5‐enolpyruvylshikimate‐3‐phosphate synthase (*epsps*), glyphosate N‐acetyltransferase (*GAT*) and Bialaphos resistance bar) (Chen *et al*., 2006; Liang *et al*., 2017; Meng *et al*., 2023; Toki *et al*., 1992) and sugars (such as xylose isomerase (*xylA*) and mannose phosphate isomerase (*pmi*)) (Guamán *et al*., 2018; Voronovsky *et al*., 2005; Wu and Zhao, 2017) can also be used for screening transformants. Furthermore, reporter genes can be added to characterize gene expression activity, enabling the determination of whether exogenous genes have been successfully transferred into plant cells, tissues or organs.

**Table 2:** Relevant markers in the process of genetic transformation

Type	Gene name	Full‐name	Phenotype	References
Reporter gene	*GFP*	Green fluorescent protein	Green fluorescent	Remington (2011)
	*eGFP*	Enhanced Green Fluorescent Protein	Green fluorescent	Li *et al*. (2014)
	*eYGFPuv*	Enhanced Yellow‐Green Fluorescent Protein like protein under ultraviolet	Green fluorescent	Yuan *et al*. (2023)
	*YFP*	Yellow Fluorescent Protein	Yellow Fluorescent	Shaner *et al*. (2004)
	*DsRed*	Discosoma striata Red	Red Fluorescent	Jach *et al*. (2001)
	*mCherry*	monmer Cherry	Red Fluorescent	Huang and Yonglun (2023)
	*GUS*	β‐glucuronidase	Hydrolyzes X‐Gluc to blue product	Jefferson *et al*. (1987)
	*Luc*	Luciferase	Bioluminescence	Azad *et al*. (2014)
	*RUBY*	Betalain	Visible Red	He *et al*. (2020)
	*FT*	Flowering Locus T	Early flowering	Putterill and Varkonyi‐Gasic (2016)
	*PAP1*	production of anthocyanin pigment 1	Visible Purple	Liu *et al*. (2019)
	*GBVS*	GL2 mutation‐based visible selection	Epidermal hair development	Kong *et al*. (2020)
Antibiotic	*nptII*	Neomycin phosphotransferase II	Kanamycin resistance/G418	Padilla and Burgos (2010)
	*hpt*	Hygromycin phosphotransferase	Hygromycin B resistance	Majumder *et al*. (2021)
	*aadA*	Aminoglycoside Adenylyltransferase	Streptomycin/Spectinomycin resistance	Saleh *et al*. (1988)
	*aac*	Aminoglycoside Acetyltransferase	Gentamycin resistance	Wang *et al*. (2019b)
	*dhps*	dihydrofolate synthetase	Sulphonamide resistance	Banerjee *et al*. (1988)
	*dhfr*	Dihydrofolate reductase	Methotrexate resistance	Blakley and Sorrentino (1998)
	*cat*	Chloramphenicol acetyltransferase	Chloramphenicol resistance	Smale (2010)
Herbicide	*epsps*	enolpyruvyshikimate phosphate synthase	Glyphosate resistance	Chen *et al*. (2006)
	*Als*	Acetolactate Synthase	Sulphonylurea resistance	Corbett and Tardif (2006)
	*bar/pat*	Glufosinate acetate transferase	Bialaphos, glufosinate resistance	Herman *et al*. (2011)
Other	*xylA*	Xylose isomerase	Grown in xylose	Barreto *et al*. (2023)
	*pmi*	Phosphomannose isomerase	Grown in mannitol	Joersbo *et al*. (2000)
	*badh*	Etaine aldehyde dehydrogenase	Detoxify toxic betaine aldehyde	Fitzgerald *et al*. (2009)
	*ipt*	Isopentenyl transferase	Grown in the absence of IAA hormone	Esmaeili *et al*. (2019)
	*hemL*	Pyridoxamine 5′‐phosphate‐dependent glutamate‐1‐semialdehyde‐2,1‐aminomutase	Detoxification of 3‐amino‐2, 3‐dihydrobenzoic acid	Nardella *et al*. (2019)
	*lysC*	Lysine‐Threonine‐Aspartate kinase	Lysine insensitive	Huang *et al*. (2019)

Wild‐type GFP, while widely used, suffers from low expression efficiency, weak fluorescence and slow chromophore formation, which limits its application as a visual reporter gene in transgenic plants (Cubitt *et al*., 1995). The mutant form, sGFP (S65T), has improved fluorescence properties and is widely used in transgenic tobacco for screening under a fluorescence microscope (Harper *et al*., 1999). Enhanced Green Fluorescent Protein (eGFP) emits over six times more fluorescence than GFP, making it more suitable for studying gene expression, regulation, cell differentiation and protein localization and transport in organisms (Duwé *et al*., 2015).

The *DsRed* gene, derived from the red fluorescent protein of *Discosoma* coral, is the first coral‐derived fluorescent protein used as a reporter gene in plants (Jach *et al*., 2001; Sun *et al*., 2018). It is one of the most widely used fluorescent proteins in biology after GFP, emitting red fluorescence under UV light. With its longer emission wavelength, superior sensitivity and signal‐to‐noise ratio compared to GFP, it is an excellent complement to GFP‐based *in vivo* studies. The eYGFPuv gene, a novel green fluorescent protein, exhibits bright fluorescence under UV light and can be visibly observed without special filters in transgenic plants. When transferred to petunia, it exhibited stable fluorescence with no attenuation, even with prolonged UV exposure (Chin *et al*., 2018).

The β‐glucuronidase (*GUS*) gene, encoded by a hydrolase enzyme that catalyses the hydrolysis of β‐glucoside esters, is often used as a reporter gene in plant genetic engineering because it does not have detectable activity in higher plants (Jefferson *et al*., 1987). GUS activity can be detected using 5‐bromo‐4‐chloro‐3‐indolyl‐glucuronide (X‐Gluc), which stains the tissue blue, visible either with the naked eye or under a microscope (Mudunkothge *et al*., 2023).

mCherry, a red fluorescent protein derived from mushroom coral, is frequently used to label and trace molecular and cellular components. Compared to other fluorescent proteins, mCherry has superior photostability and can be co‐labelled with GFP for dual‐colour imaging. In a CRISPR/Cas9‐based screening system, mCherry fluorescence helps identify T‐DNA‐free and edited seeds by selecting red‐fluorescent T1 seeds and non‐fluorescent T2 seeds (Gao *et al*., 2016; He and Zhao, 2019).

Betalain, a natural product synthesized from tyrosine by three enzymes (CYP76AD1, DODA and GT), imparts the bright red colour seen in beets, dragon fruits and Swiss chard (Polturak and Aharoni, 2019). By inserting the coding sequence for these enzymes into a binary vector T‐DNA, betalain production can be used to visually identify transgenic positive callus or plants (He *et al*., 2020). This method eliminates the need for chemical treatments or specialized equipment, offering an alternative for large‐scale plant transformations where antibiotic or herbicide screening is impractical.

FT (Flowering Locus T) has been identified as encoding a mobile signalling molecule that triggers early flowering in plants (Putterill and Varkonyi‐Gasic, 2016). Thus, FT expression can potentially accelerate the selection of gene‐edited plants. The overexpression of the anthocyanin synthesis gene PAP1 leads to a distinct purple coloration in the leaves of various plant species (Zhang *et al*., 2014). A novel gene editing vector, Cas9‐PF, was developed by integrating both the PAP1 gene and FT into the CRISPR/Cas9 system. Transformants with low transformation or expression efficiency, identifiable by green or purple‐green leaf colour, were excluded through visual leaf colour observation. In contrast, transgenic seedlings exhibiting efficient CRISPR/Cas9 editing were selected for further study (Liu *et al*., 2019).

## Prospect

### Explant selection for plant tissue culture

The selection of explants for plant tissue culture is crucial for successful genetic transformation. Variations in transformation efficiency can arise from differences in species, genotypes, explant types and the developmental stages of recipient materials. These factors contribute to the variability and instability observed in genetic transformation studies. Research on the developmental status of cells and transformation efficiency in various recipient materials has revealed that explants collected during the optimal transformation period exhibit specific morphological and cytological characteristics. These explants show the highest proportion of cells in the S phase, as detected by EdU staining and flow cytometry. Additionally, the key genes *CDKB1*, *CDKD1* and *CYCD6* in the S phase, identified through quantitative PCR, are associated with peak adventitious bud primordium cell formation. These findings suggest that genetic transformation is most efficient and stable when applied at this critical developmental window (Xia *et al*., 2023).

Improving crop regeneration efficiency remains a significant focus of plant tissue culture and cell engineering. The regenerative abilities of certain animals, such as geckos, starfish, zebrafish and earthworms, offer interesting parallels. For instance, starfish can regenerate entire individuals from small fragments (Ben Khadra *et al*., 2015; Marques *et al*., 2019). However, both animal and plant regeneration capacities diminish with age. In plant genetic transformation, young embryos are commonly chosen as explants and regeneration typically occurs from seedlings. For example, the shoot apical meristem cell‐mediated transformation method (SAMT) has proven effective in transforming cotton, offering valuable support for breeding efforts (Ge *et al*., 2023). However, adult explants of many plants struggle to form calluses capable of differentiation when exposed to hormonal treatments. Moreover, the regenerative potential of calluses declines with prolonged subculturing, yet the molecular mechanisms behind this phenomenon remain unclear. Understanding these processes will aid in deciphering how age influences plant cell fate and regeneration potential, thereby enabling the development of more efficient regeneration systems.

### Pollen grain‐mediated transformation

Pollen plays a critical role in plant reproduction by transferring male gametes through cross‐fertilization, thus contributing to genetic variation. The success of fertilization, embryogenesis and seed development depends on the pollen's ability to remain viable long enough to deliver male gametes to the awaiting ovules. In 1990, it was discovered that pollen could be used as a DNA vector (Alwen *et al*., 1990; Kanwal *et al*., 2022). Genetic modification of maize was achieved by irradiating pollen (Pandey, 1975) and by complexing DNA with pollen prior to using the treated pollen for fertilization (Ohta, 1986). Recent advances have enhanced this approach, with the use of DNA‐coated magnetic nanoparticles to significantly improve pollen germination pore opening, all while maintaining pollen viability. Through this method, exogenous genes can be introduced into pollen via germination pores, utilizing nanobeads, opening up new possibilities for pollen‐mediated genetic transformation (Wang *et al*., 2022a, 2022b, 2022c; Zhao *et al*., 2017).

The success of using pollen as a DNA vector largely depends on the pollen's viability after treatment, as well as the high endogenous sucrose content before *in vitro* culture. The sucrose level in the medium following pollen separation may exert pressure on the pollen wall (Ahmad and Martin, 2017; Mbogning *et al*., 2007). Furthermore, factors like drying or desiccation during anther division can significantly reduce pollen viability (Jayaprakash *et al*., 2015). Temperature is also a key factor influencing pollen vitality (Müller and Rieu, 2016; Santiago and Sharkey, 2019). Additionally, the morphological characteristics of pollen, such as size, shape, aperture number and aperture size, can affect its suitability as a DNA vector.

### Malformations and sterility in genetic transformation

Tissue culture has led to unprecedented advancements in genetic engineering, but it often results in the generation of malformed seedlings and the occurrence of malformations and abortion in transgenic materials. These issues can hinder the development and application of plant tissue culture technologies (Bernard *et al*., 2007; Loyola‐Vargas and Ochoa‐Alejo, 2018; Zuzarte *et al*., 2024). Among the various factors contributing to malformed seedlings, the formation of deformed embryoid bodies is the primary cause. Once a malformed embryoid body forms, it is challenging to induce normal seedlings directly, and most will develop into malformed seedlings. Therefore, controlling the formation of malformed embryoid bodies is a critical step. Additionally, since malformed seedlings are products of artificially provided media and culture conditions, the culture environment significantly influences their occurrence. The incidence of malformed seedlings also varies based on genetic backgrounds (Espinosa‐Leal *et al*., 2018; Palee *et al*., 2012). Many researchers have linked the formation of malformed seedlings to factors such as hormones, sugars, moisture, curing agents, culture temperature and culture containers in the culture medium (Sharma *et al*., 2023).

Malformed embryoid bodies have been observed in anther culture, with variations noted at all stages of cotton somatic cell culture, from cells to regenerated plants. Different types of malformed embryoid bodies can be seen through sectional observation and external morphology (Zhang *et al*., 1993). Regenerated plants may exhibit defects such as regeneration failure, albinism, male sterility and embryo abortion, which are influenced by gene regulation. Male sterility is induced by the CMS (cytoplasmic male sterility) gene, while embryo abortion is linked to differentially expressed genes (DEGs) and proteins (DEPs) in the regenerated plants (Asaduzzaman *et al*., 2022; Sharma *et al*., 2023). Moreover, plant tissues are cultivated in a closed, sterile environment, representing a unique environmental stress for plants. High morphological growth and developmental processes are induced in tissue‐cultured plants, with specific regulatory genes driving morphogenesis in this controlled environment (Yin *et al*., 2022).

Previous studies have analysed influencing factors in isolation, but plants are complex, interconnected organisms. The absorption and transformation of substances within the plant are interrelated. The impact of *in vitro* substances on the changes occurring within the plant body, and how these changes affect other substances, is crucial to understanding plant development. Exogenous hormone concentration changes can alter callus morphology, and when plants absorb these hormones, they also synthesise endogenous hormones. Understanding how exogenous and endogenous hormones interact and how these hormonal changes regulate callus morphology is key to addressing the issue of malformed seedlings. However, in‐depth research is lacking in this area. Organ regeneration in tissue culture is influenced by multiple factors, and the formation of malformed seedlings results from the combined action of these factors. Modifying some of these factors may lead to desired outcomes. Understanding the mechanisms behind malformed seedling formation and minimising their occurrence is essential for advancing plant tissue culture research, transgenic research and molecular biology applications.

### Functional conservation of regeneration‐related factors

Over 20 regenerative factors have been identified, but their functions are not conserved across different species. For example, *BBM‐WUS2* has been widely used in maize and has achieved high transformation efficiency in many previously non‐transformable maize inbred lines. However, it has minimal effect on some dicots, such as cotton. Additionally, ectopic expression of BBM/WUS2 can negatively impact the growth of regenerated plants and induce sterility (Lowe *et al*., 2016). Co‐transformation of GRF proteins and their co‐factor GIF significantly improved regeneration and transformation efficiencies in wheat, triticale and rice, showing notable success in monocots but limited effects in dicots (Debernardi *et al*., 2020). The *TaWOX5* gene, similar to GRF‐GIF, addresses the genotype‐dependent issues in wheat genetic transformation, enhancing transformation efficiency. This gene holds great potential for genetic transformation in cereal crops (Wang *et al*., 2022a, 2022b, 2022c). In plants, *WUSCHEL HOMEOBOX (WOX)* genes are crucial regulators of plant growth and development, particularly through their control over the production and maturation of meristem stem cells (Hendelman *et al*., 2021). While *WOX* homologues are conserved in their meristem functions across different plants, their roles can vary. In *Arabidopsis thaliana*, mutations in the *AtWOX8* and *AtWOX9* genes cause embryogenesis defects (Breuninger *et al*., 2008). Tomato has a *WOX8/9* homologue, and mutations in this gene result in over‐branching of the inflorescence (Lippman *et al*., 2008). In related species, such as pepper and petunia, *WOX8/9* mutations lead to stem meristem hyperplasia without flowering. In rice, the *WOX8/9* mutation causes hyperplasia of lateral branch meristems, shortening of internodes and altered inflorescence branching (Wang *et al*., 2014).

The non‐conserved functions of these homologous genes across species create uncertainty for the widespread use of regenerative factors. Therefore, identifying and improving regenerative factors to overcome functional inconsistencies between crops will be crucial for advancing breeding engineering.

### Emerging sequencing strategies for screening regenerative genes

Single‐cell RNA sequencing (scRNA‐seq) has revolutionized the study of cellular heterogeneity in animals but remains underutilized in plant research. Nevertheless, its advantages at the single‐cell level make it highly suitable for exploring somatic cell regeneration in plants. The first application of the 10x Genomics single‐cell sequencing platform in plants produced 7552 single‐cell transcriptomes from *Arabidopsis* root protoplasts, demonstrating the feasibility and utility of scRNA‐seq in plant studies (Ryu *et al*., 2019). Subsequent research captured transcriptomes from 4727 single cells derived from *Arabidopsis* root tissues, providing insights into key developmental regulators and downstream genes involved in cell fate transitions. Using pseudotime analysis, researchers traced the lineage trajectories of root hair cells, identifying novel genes critical for root hair differentiation (Denyer *et al*., 2019). Building on this foundation, single‐cell transcriptome sequencing was performed on apical cells of two rice subspecies, Japonica Nip and Indica 93‐11, mapping the developmental trajectories of outer root cells (Liu *et al*., 2021).

Advancing these studies further, a spatial transcriptomic map of plant callus bud regeneration was constructed by integrating spatial transcriptome technology with single‐cell nuclear RNA sequencing. This breakthrough revealed the heterogeneity within tomato calli, categorizing cells into five types—epidermis, vascular tissue, bud primordium, mature bud and internal callus—and identified key regulators of bud primordium formation (Song *et al*., 2023).

Real‐time imaging and scRNA‐seq have provided further insights into plant regeneration. For example, the transcription factors WUS and DRN were identified as essential for regenerating *Arabidopsis thaliana* mesophyll cells, promoting the reprogramming of these cells (Xu *et al*., 2021). Additionally, Quartz‐Seq2 analysis highlighted the role of *WOX13* in shaping callus cell identity. *WOX13* negatively impacts bud regeneration efficiency by driving cell differentiation toward highly expansive spheroid cells, while antagonizing key meristem regulators such as *WUS*, *STM*, *ESR2* and *CUC1*. These findings suggest that *WOX13* and *WUS* form a mutually inhibitory regulatory circuit critical for regeneration efficiency (Ogura *et al*., 2023).

In wheat, RNA‐seq and ATAC‐seq were used to construct a transcriptional regulatory network. The study uncovered differences in the transcription factor families activated during early callus induction in wheat and *Arabidopsis*: the DOF and G2‐like families dominated in wheat, while the NAC and LBD families were prominent in *Arabidopsis*. Overexpression of NAC and LBD family members enhanced genetic transformation in *Arabidopsis*, and a similar effect was observed with DOF family transcription factors in wheat (Liu *et al*., 2023). In cotton, comparative analyses of regenerative and non‐regenerative materials revealed key genes responsible for somatic embryogenesis. Using single‐cell transcriptomics, researchers constructed a gene regulatory network for cotton somatic embryogenesis, providing critical insights into the regeneration process (Zhu *et al*., 2023a).

Unlike higher animals, plant cells exhibit remarkable plasticity, allowing differentiated somatic cells to regain totipotency. While many plant species have successfully regenerated from single somatic cells, the underlying mechanisms remain poorly understood. The integration of single‐cell sequencing and multi‐omics analyses offers a powerful strategy for elucidating cell totipotency mechanisms. These findings not only advance plant regenerative biology but also hold potential implications for mammalian stem cell research and somatic cell reprogramming.

The rapid evolution of sequencing technologies has significantly advanced research on crop regeneration efficiency, progressing from ‘single‐cell transcriptome technology’ to ‘single‐cell multi‐omics technology’ and, more recently, to ‘spatial transcriptome technology (Spatial Transcriptomics, ST)’ (Chen *et al*., 2021; Rao *et al*., 2021; Zormpas *et al*., 2023). Single‐cell transcriptome sequencing has enhanced the understanding of gene expression at the single‐cell level, proving particularly useful in discovering new cell types and revealing cellular heterogeneity. However, the enzymatic hydrolysis required to obtain single‐cell suspensions can result in the loss of spatial positioning information of the cells, causing gene expression profiles to change during or after the enzymatic process. Additionally, some specialized cell forms are difficult to isolate via tissue enzymatic hydrolysis.

ST technology, which captures both spatial location and gene expression data simultaneously, addresses these limitations by allowing the study of *in situ* gene expression in tissue cells without the need to prepare cell suspensions. This provides a valuable tool for research in various fields, such as tissue cell function, microenvironment interactions, developmental lineage tracking and disease pathology (Wang *et al*., 2023b). In plant biology, ST technology enables the identification of cell types, the construction of cell fate and developmental lineages and the analysis of cell‐to‐cell communication and interactions (Rao *et al*., 2021).

Overall, the single‐cell resolution and broad field of view offered by ST technology are ushering in a new era of plant research. By revealing spatially specific transcriptome signatures and decoding cell‐to‐cell communications, it provides new insights into plant biology. In the future, combining ST with other spatial omics approaches will further enhance our understanding of plant development, metabolism and microbiota interactions (Yin *et al*., 2023).

## Funding

The authors thank Biological Breeding‐National Science and Technology Major Project (2024ZD0407704), Nanfan special project, CAAS (YBXM2415), National Natural Science Foundation of China Youth Science Fund (32201856), Hebei Key Technology R&D program (21326314D) and Winall Hi‐tech Seed Co., Ltd. (GMLM2023) for their support.

## Conflict of interest

The authors declare no competing interests.

## Authors' contributions

Conceptualization, WP, SH, LC and XZ; writing original draft preparation, WP; review and editing, GH, JS and CH; funding acquisition, XZ, GH and CH. All authors have read and agreed to the published version of the manuscript. All authors read and approved the final manuscript.

## Data Availability

Data availability is not applicable to this article as no new data were created or analysed in this study.
